# Pseudoamblyopia in Congenital Cyclotropia

**DOI:** 10.1155/2017/1870290

**Published:** 2017-07-26

**Authors:** Antonio Frattolillo, Filippo Tassi, Valentina Di Croce, Costantino Schiavi

**Affiliations:** ^1^Department of Ophthalmology, S. Martino Hospital, Viale Europa, 22, 32100 Belluno, Italy; ^2^Department of Experimental, Diagnostic, and Specialty Medicine (DIMES), Ophthalmology Service, S.Orsola-Malpighi Teaching Hospital, University of Bologna, Via P. Palagi, 9, 40138 Bologna, Italy

## Abstract

**Purpose:**

To study the effect of surgery on amblyopia and suppression associated with congenital cyclovertical strabismus.

**Methods:**

The fixation pattern was investigated with microperimetry before and soon after surgery in ten consecutive children operated for congenital superior oblique palsy at the S. Martino Hospital, Belluno, Italy, between September 2014 and December 2015. Changes in visual performance in terms of best-corrected visual acuity (BCVA) and stereopsis between the day before and one week after surgery were also evaluated. No other amblyopia treatment has been administered during the time study.

**Results:**

Surgical correction of the excyclodeviation in congenital SO palsy determined monocular and binocular sensory consequences: monocularly, in the cyclodeviated amblyopic eye, BCVA (0.46–0.03 LogMAR; *p* < 0.0001) and the fixation pattern improved, as demonstrated by microperimetry examination. Binocularly, stereopsis improved or emerged while suppression at the Worth four-dot test disappeared.

**Conclusions:**

In the absence of further amblyopic factors such as coexisting constant vertical and/or horizontal deviation and anisometropia, the amblyopia encountered in congenital SO palsy may resolve soon after the surgical alignment. Therefore, it may be considered and defined “pseudoamblyopia.”

## 1. Introduction

Cyclodeviations are special forms of strabismus characterized by a misalignment of the eyes around the line of sight, that is, the anteroposterior axis. Cyclodeviations rarely exist as an isolated disturbance of ocular motility while they are often associated with paralysis of the oblique or vertical rectus muscles [1]. Superior oblique (SO) muscle palsy is the most common cause of cyclotropia and may be congenital or acquired. In SO palsy, a vertical deviation usually coexists which may be compensated with an anomalous head posture. Pure cyclotropia is also generally compensated by a head tilt toward the side of the incyclodeviated eye or the opposite side in case of excyclotorsion of one eye.

SO palsy causes a vertical deviation in the field of action of the underacting SO and overacting inferior oblique with excyclotorsion of the affected eye. Patients with unilateral SO palsy usually show hyperdeviation of the affected eye that decreases from adduction to primary position and abduction and a compensatory head posture consisting in a head tilt and face turn to the contralateral side.

In acquired palsies, the 4th cranial nerve or nucleus can be damaged from blunt head trauma or vascular ischemic accidents. In recently acquired palsies, there is a characteristic motility pattern with hyperdeviation and excyclotorsion of the affected eye in down gaze [2]. In acquired cyclodeviations, besides vertical diplopia, patients commonly experience image tilting.

Congenital SO muscle palsy is found since early infancy in children presenting with abnormal head posture, that is, compensatory head tilt to the contralateral nonparetic side, without any obvious determining cause of the paralysis [2]. The congenital SO palsy is suspected to have a genetic background, as pointed out by familial occurrence [3], evidence on MRI of superior oblique muscle and/or tendon hypoplasia or absence [4, 5] or detection of single nucleotide polymorphisms (SNPs) of the genes expressed in the trochlear nucleus of the brain stem [6]. In congenital SO palsy, the most evident ocular motility pattern is overaction of the inferior oblique muscle.

In congenital cases, patients usually do not perceive image tilting for either binocular or monocular sensory compensatory mechanisms. Binocular sensory adaptation consists of suppression and/or anomalous retinal correspondence. Monocular sensory adaptation consists of spatial reorientation of the horizontal and vertical retinal meridians along new meridians.

Torsional eye movements consist of reflex movements driven by the vestibulo-ocular system and cyclovergence movements driven by the vergence system. Torsional versions are involuntary movements evoked by the vestibulo-ocular system during head roll. The torsional vestibulo-ocular reflex (VOR) generates conjugate torsional eye movements which consist of excyclotorsion of one eye and incyclotorsion of the other that compensate for head tilt. While cycloversion concerns conjugate torsional movements of the eyes provoked via vestibular stimulation, cyclovergence refers to counterrotations between the two eyes whose aim is to compensate for cyclotorsion during ocular movements in order to maintain fusion.

These binocular and monocular sensory adaptations to cyclotropia that occur in patients with congenital or early acquired cyclodeviation [1] prevent awareness of tilting of the environment under monocular and binocular conditions. Such spatial adaptation compensates for the image tilt that would otherwise be perceived. It explains why patients with objective cyclotorsion of one eye continue to see a vertical line as vertical and a horizontal line as horizontal in the absence of all other visual clues. This complete sensory adaptation may be facilitated by the tendency of patients with congenital onset SO palsy to have smaller degrees of objective cyclotropia than those with acquired cyclotropia. However, this complete adaptation is not irreversible [1]. Patients with congenital cyclodeviation may momentarily note tilting of environment in the opposite direction after surgery for correcting cyclotropia before the innate, normal spatial orientation of retinal meridians reestablishes itself [7]. These binocular and monocular sensory adaptations may not prevent the onset of amblyopia. In fact, in congenital SO palsy, amblyopia and subnormal stereopsis are common clinical findings.

There is clinical evidence of increased prevalence of amblyopia in children presenting with congenital SO palsy and overaction of the inferior oblique muscle with or without abnormal position of the head. Usually amblyopia affects the eye with the SO deficit but the opposite may happen. The occurrence of amblyopia in these cases usually is combined with absence of normal binocular vision and random dot stereopsis [8, 9].

Basing on the clinical observation of sudden improvement of visual acuity and stereopsis in children operated on for congenital SO palsy, as if this kind of amblyopia was only apparent or quickly reversible and soluble by the surgical correction of cyclotropia, we studied in deep a series of patients who had undergone inferior oblique recession for congenital SO palsy at the Ophthalmology Department of the S. Martino Hospital of Belluno, north Italy. Besides a full ophthalmological and orthoptic evaluation, the study included microperimetry assessment of the fixation pattern of the amblyopic eye before and after surgery.

## 2. Patients and Methods

This study includes ten consecutive children aged 4 to 9 years (mean 5.6 years, standard deviation 1.684), 6 females and 4 males, operated for congenital SO palsy at the S. Martino Hospital of Belluno from February 2014 to December 2015. Diagnosis of congenital SO palsy was confirmed on ocular motility examination by the presence of characteristic SO underaction (SOUA), inferior oblique overaction (IOOA), positivity of Bielschowsky head tilt test, and compensatory head tilt toward the sound side.

All patients showed small-angle hypertropia, that is, ≤12^Δ^ in primary position of gaze with the head straight, and were near orthotropia with the head tilted toward the sound side. However, most of them did not display stereo visual acuity.

Refractive errors ranged from −1.50 to +2.75 of spherical equivalent (SE), and anisometropic error did not exceed 0.50 diopters SE in any patient. Patients with astigmatism higher than 0.5 diopters in the eye with the superior oblique deficit were excluded in order to avoid a refractive effect on postoperative visual acuity resulting from the rotation of the axis of astigmatism induced by surgery. We did not also include patients with the paretic eye fixating and the other amblyopic, since in this condition, it is quite probable that a current or previous anisometropia has promoted fixation of the paretic eye, and the aim of the present study was to investigate strabismic and not anisometropic amblyopia.

Cycloplegic refraction was determined with retinoscopy in either the preoperative or postoperative visit.

Also, photographic or historical documentation of a head tilt since early childhood and/or the presence of facial asymmetry were considered as confirming clinical signs.

The inclusion criteria were the presence of congenital SO palsy, ability to cooperate for subjective and objective torsional measurements and microperimetry assessment, and no history of strabismus surgery or ocular pathologies, apart from SO palsy.

Patients' parents gave informed consent after the purpose of this study was explained, and the procedures conformed to the tenets of the Declaration of Helsinki.

All patients underwent comprehensive ophthalmological and orthoptic examination the day before and 7 days after surgery. Ophthalmological examination included best-corrected visual acuity (BCVA) evaluation that was measured at a distance of 5 meters with “E” charts and microperimetry examination. Orthoptic evaluation consisted of ocular motility examination, Worth's four-light test, and stereoacuity measurement. Parks' 3-step test [10] was combined with the prism cover/uncover test to identify the underacting cyclovertical muscle by recording the vertical deviation in primary, left and right gaze with the head tilted to the right and to the left. Stereoacuity was measured with either Lang stereotest (Lang-Stereotest AG, Küsnacht, Switzerland) in all patients or TNO test (Lameris Instrumenten B.V., Utrecht, The Netherlands) in 6 out of 10 patients.

MP-1 microperimetry was used to assess fixation stability pattern of the deviated eye. Responses were scaled in the following fashion [11]:

(1) stable, if >75% of fixation points fall within a 2° diameter circle centered on the gravitational center of all fixation points; (2) relatively unstable, if <75% of fixation points fall within a 2° circle but >75% are located within a 4° diameter circle; and (3) unstable, if <75% of all fixation points fall within a 4° diameter circle.

For the MP-1 examination, the fixation target was the standard 2° diameter red cross, set against a dim white background with a luminance level of 1.27 cd/m2; the range of Δ*L* (luminance difference between stimulus and background) was 1.27 to 127 cd/m2 producing a dynamic range of 0–20 dB; stimulus size was Goldmann III; duration was 200 ms; and testing protocol was 4-2 threshold strategy. The standard 10-2 grid was used consisting of 68 test loci arranged in a Cartesian pattern covering the central 20°.

BCVA was measured with the cycloplegic optical correction the day before surgery and at the follow-up visit carried out one week after surgery. No orthoptic or amblyopia treatment was given in the time elapsed from surgery and the follow-up visit. No change in the optical correction was prescribed in the same time.

All operations were performed under general anesthesia by the same surgeon (AF). All patients underwent IO graded recession in the affected eye. The amount of recession ranged from 5 to 10 mm, according to this simple formula based on the size of the latent or manifest hyperdeviation in primary position of gaze with the head straight: 5 to 7 mm recession for hyperdeviations ranging from 1 to 4 prism diopters (PD), 8 to 9 mm recession for hyperdeviations ranging from 5 to 9 PD, and 10 mm recession for hyperdeviations of 10 to 12 PD.

## 3. Statistical Analysis

Comparisons between preoperative and postoperative BCVA values were statistically analyzed using the Wilcoxon rank sum test. Also, changes in refractive errors induced by surgery were statistically analyzed with the Wilcoxon rank sum test. Statistical analyses were performed using SPSS (SPSS, Chicago, Illinois, USA), and *p* value of <0.05 was considered statistically significant.

## 4. Results

The summary of pre- and postoperative measurements of BCVA, refraction, stereopsis (Lang test and TNO test), and Worth 4-light test is shown in Table 1.

Mean BCVA of the operated eyes was 0.46 ± 0.16 LogMAR before surgery and 0.03 ± 0.04 LogMAR after surgery, and the difference was statistically significant (*p* < 0.0001). Improvement in BCVA after surgery was recorded in 100% of the operated eyes (Table 1).

Cycloplegic refraction of the operated eyes, as determined with retinoscopy in either the preoperative or postoperative visit, showed a mean SE value of 0.35 ± 1.36 before surgery and 0.35 ± 1.27 after surgery, and the difference was not statistically different (*p* = 0.940) (Table 2). No refractive changes were found in the other eye in any patient.

Before surgery BCVA was 0.0 LogMAR in the sound eye of every patient and no change was observed after surgery.

The Lang test showed absence of stereopsis in 100% of the patients before surgery and presence of stereopsis in 100% of them after surgery. After-surgery stereopsis in every patient was 550 seconds of arc.

Random dot stereopsis measured with the TNO test in 6 out of 10 patients resulted absence in all the measured patients before surgery. After surgery, 4 out of 6 patients showed random dot stereopsis.

All the patients exhibited suppression of the eye with SO palsy before surgery (three or two lights seen at Worth's four-light test), and none of them complained of diplopia. After surgery, all the patients saw four lights at Worth's four-light test.

Microperimetry examination gave the following results: 8 patients who showed unstable fixation before surgery were classified as relatively unstable after surgery. In the remaining 2 who had showed relatively unstable fixation before surgery, fixation remained unchanged after surgery. Before surgery, all patients presented predominantly eccentric fixation. After surgery, 8 of them had poor central fixation and 2 predominantly central fixation (Table 3).

## 5. Discussion

The oblique extraocular muscles play a predominant role in vertical fusional vergence in healthy individuals [12, 13]. Prism-induced vertical disparity in healthy subjects can produce vertical fusional vergence, and then, cycloversion of eyes occurs, with the downward-moving eye intorting and the upward-moving eye extorting [12]. This movement pattern was confirmed in subsequent studies using afterimage techniques [14], and automated video-oculography [11] further supported the idea that the oblique muscles were the primary mediators of normal vertical fusional vergence. Dysfunction of oblique muscles induces a misalignment of the eye on the anteroposterior axis. This can induce torsional diplopia or image tilting.

Patients with congenital SO palsy are commonly asymptomatic since cyclodeviation is compensated by cyclofusion through cyclovergence and by sensory monocular and binocular adaptations. Whether cyclofusion occurs only on a sensory basis or has also a motor component, and which weight has the one and the other in compensating for cyclotropia, is still a debated question. Anyway, in congenital SO palsy, cyclofusion and/or such sensory adaptations, even if compensate for image tilting, may not prevent the onset of amblyopia and loss of stereopsis. However, this kind of amblyopia which occurs in congenital SO palsy may be presumed to be less severe and faster resolvable than that encountered in other forms of strabismus. In fact, in congenital SO palsy, the cyclovertical deviation may be compensated, at least in some positions of gaze, with the patient maintaining the head tilted on the sound side. It can be supposed that in these circumstances, in absence of concurrent vertical and/or horizontal deviations in some position of gaze and in absence of anisometropia, the degree of foveal suppression of one of the two eyes is not deep, probably because the two visual axes optically coincide and the misalignment between them is only torsional. The sudden improvement of the fixation pattern and visual acuity of the amblyopic eye and the increase or onset of stereopsis in response to surgical correction of the cyclovertical deviation in our case series could be explained with the presence of such not deeply rooted “torsional” amblyopia that is caused by the torsional misalignment of the fovea of the deviated eye. Likewise, the sudden improvement or onset of stereopsis after surgery in such cases could be explained with the presence of an inhibited rather than absent stereopsis. Surgical correction of the cyclodeviation in congenital SO palsy determines monocular and binocular consequences: monocularly, in the cyclodeviated amblyopic eye, visual acuity increases and the fixation pattern improves, as demonstrated by microperimetry examination; binocular stereopsis improves or emerges, while suppression disappears. In the absence of further amblyopic factors such as coexisting vertical and/or horizontal deviations and anisometropia, the amblyopia encountered in congenital SO palsy may be therefore defined “pseudo” amblyopia, since it may resolve soon after the surgical correction of the torsional deviation, without any other optical or orthoptic treatment.

Our study has some limitations that may affect its scientific significance. In fact, we presume that the improvement in visual acuity and stereopsis is due to the surgical intervention. However, there is no control group, no placebo group, and no comparison to other treatment interventions (e.g., occlusion, prism, and orthoptic vision therapy).

Nevertheless, it seemed appropriate to point out this clinical observation and speculate on the possible explanations.

## Figures and Tables

**Table 1:** Summary table.

Patient	Age	BCVA pre	BCVA post	SE pre	SE post	Lang pre	Lang post	Worth pre	Worth post	TNO pre	TNO post
1	8	0.5	0	−1.00	−1.00	Absent	550″	Suppression	SBV	NE	NE
2	9	0.4	0.1	0.38	0.50	Absent	550″	Suppression	SBV	NE	NE
3	6	0.5	0	1.50	1.13	Absent	550″	Suppression	SBV	NE	NE
4	5	0.3	0	1.13	1.13	Absent	550″	Suppression	SBV	NE	NE
5	4	0.2	0	2.75	2.50	Absent	550″	Suppression	SBV	Absent	Plate II
6	4	0.5	0	−1.25	−1.13	Absent	550″	Suppression	SBV	Absent	Plate III
7	6	0.3	0	−1.50	−1.38	Absent	550″	Suppression	SBV	Absent	Absent
8	5	0.7	0.1	0.50	0.75	Absent	550″	Suppression	SBV	Absent	Plate V
9	4	0.5	0	1.75	1.75	Absent	550″	Suppression	SBV	Absent	Absent
10	5	0.7	0.1	−0.75	−0.75	Absent	550″	Suppression	SBV	Absent	Plate V
Mean		0.46	0.03	0.35	0.35						
SD		0.16	0.04	1.37	1.27						
*p* value^∗^		0.0001		0.94							

SD = standard deviation; SBV = single binocular vision; SE = spherical equivalent; ″ = seconds of arc; NE = not evaluated; ^∗^Wilcoxon test pre- and post-BCVA.

**Table 2:** Refractive error.

Pre-OP			Post-OP		
SPH	CYL	SE	SPH	CYL	SE
−1.00		−1.00	−1.00		−1.00
0.50	−0.25	0.38	0.75	−0.50	0.50
1.50		1.50	1.25	−0.25	1.13
1.00	0.25	1.13	1.00	0.25	1.13
2.75		2.75	2.50		2.50
−1.25		−1.25	−1.25	0.25	−1.13
−1.25	−0.50	−1.50	−1.25	−0.25	−1.38
0.50		0.50	0.75		0.75
1.75		1.75	1.75		1.75
−0.75		−0.75	−0.75		−0.75

SPH = sphere; CYL = cylinder; SE = spherical equivalent.

**Table 3:** Microperimetry results.

Fixation	Before surgery	After surgery
Unstable	8	0
Relatively unstable	2	10
Stable	0	0
Predominantly eccentric	10	0
Poor central	0	8
Predominantly central	0	2
